# Recruitment of racial/ethnic minority older adults through community sites for focus group discussions

**DOI:** 10.1186/s12889-017-4482-6

**Published:** 2017-06-09

**Authors:** Mary E. Northridge, Michele Shedlin, Eric W. Schrimshaw, Ivette Estrada, Leydis De La Cruz, Rogelina Peralta, Stacia Birdsall, Sara S. Metcalf, Bibhas Chakraborty, Carol Kunzel

**Affiliations:** 1 Department of Epidemiology & Health Promotion, New York University College of Dentistry, 433 First Avenue, 7th Floor, Room 726, New York, NY 10010 USA; 2 Department of Sociomedical Sciences, Columbia University Mailman School of Public Health, New York, NY USA; 3 Columbia University College of Dental Medicine, Section of Population Oral Health, New York, NY USA; 4 Department of Geography, The State University of New York at Buffalo, Buffalo, NY USA; 5 New York University Rory Meyers College of Nursing, New York, NY USA; 6 Duke-National University of Singapore (Duke-NUS) Medical School, Centre for Quantitative Medicine, Singapore, Singapore

**Keywords:** African Americans, Hispanics, Aging, Recruitment strategies, Health equity, Implementation science, Social science

## Abstract

**Background:**

Despite a body of evidence on racial/ethnic minority enrollment and retention in research, literature specifically focused on recruiting racially/ethnically diverse older adults for social science studies is limited. There is a need for more rigorous research on methodological issues and the efficacy of recruitment methods. Cultural obstacles to recruitment of racial/ethnic minority older adults include language barriers, lack of cultural sensitivity of target communities on the part of researchers, and culturally inappropriate assessment tools.

**Methods:**

Guided by the Consolidated Framework for Implementation Research (CFIR), this study critically appraised the recruitment of racial/ethnic minority older adults for focus groups. The initial approach involved using the physical and social infrastructure of the ElderSmile network, a community-based initiative to promote oral and general health and conduct health screenings in places where older adults gather, to recruit racial/ethnic minority adults for a social science component of an interdisciplinary initiative. The process involved planning a recruitment strategy, engaging the individuals involved in its implementation (opinion leaders in senior centers, program staff as implementation leaders, senior community-based colleagues as champions, and motivated center directors as change agents), executing the recruitment plan, and reflecting on the process of implementation.

**Results:**

While the recruitment phase of the study was delayed by 6 months to allow for ongoing recruitment and filling of focus group slots, the flexibility of the recruitment plan, the expertise of the research team members, the perseverance of the recruitment staff, and the cultivation of change agents ultimately resulted in meeting the study targets for enrollment in terms of both numbers of focus group discussions (*n* = 24) and numbers of participants (*n* = 194).

**Conclusions:**

This study adds to the literature in two important ways. First, we leveraged the social and physical infrastructure of an existing program to recruit participants through community sites where older adults gather. Second, we used the CFIR to guide the appraisal of the recruitment process, which underscored important considerations for both reaching and engaging this underserved population. This was especially true in terms of understanding the disparate roles of the individuals involved in implementing and facilitating the recruitment plan.

## Background

As the older adult population in the United States expands and diversifies more rapidly than at any other time, [1] improved understanding of the health needs of older adults is at the forefront of social science and health research [2]. Yet, the recruitment of racial/ethnic minority older adults is often a challenging and resource intensive undertaking [3, 4]. Creativity, adaptability, and targeted strategies are critical to ensuring that this underserved population is successfully recruited toward improving population health and health equity [5].

Although there are shared and distinct barriers to participation in research across racial/ethnic groups, [6, 7] lack of trust is frequently identified as a key barrier to recruitment [8]. This may be rooted in a general mistrust of mainstream society due to historical oppression and persistent discrimination, and a particular mistrust of the medical system [9]. Racial/ethnic minority older adults may demonstrate mistrust through refusal to participate, hesitancy in completing questionnaires, or hostility toward researchers [10].

One reported strategy for establishing trust with potential participants is through investigator and staff participation in community events, as exemplified by a report of playing Spanish bingo with members at a Hispanic senior center [9–11]. A second strategy involves understanding the reservations of potential participants and addressing them through culturally appropriate methods of establishing rapport and demonstrating respect. For instance, clear and repeated messages may be needed to assure Latino immigrant communities who fear institutional contacts that the information to be obtained will not place them at risk of harm [12]. A third strategy is to partner with community members and organizations, and may be accomplished by including community members in the research team or establishing a community advisory board, as per community-based participatory research approaches [13–17]. Finally, researchers have detailed the importance of establishing relationships with community gatekeepers, including clergy, community leaders, and senior center directors, to establish trust with community members and abet recruitment efforts [4, 5, 9–11, 18–20].

Cultural obstacles to recruitment of racial/ethnic minority older adults include language barriers, lack of cultural sensitivity of target communities on the part of researchers, and culturally inappropriate assessment tools [10, 21]. Racial/ethnic minority older adults may also have concerns about prejudice, racial profiling, and immigration status issues [14, 15]. These cultural barriers have been addressed through enhanced understanding of the history of the group being studied by researchers and explicitly incorporating culturally sensitive techniques into recruitment strategies [4, 9, 15, 20]. Culturally tailored approaches to recruitment that place emphasis on personal relationships may include using *personalismo* (meaning formal friendliness) and *respeto* (meaning respect) or demonstrating respect through professional attire and use of formal address, i.e., Mrs. Last Name [14]. Allowing for social conversation before initiation of research protocols may also facilitate recruitment [20].

Although there is a growing literature on minority enrollment and retention in research, [8] literature specifically focused on racially/ethnically diverse older adults is limited, with a clear need for more rigorous research on methodological issues and the efficacy of recruitment methods in this population [22]. The Consolidated Framework for Implementation Research (CFIR) provides a menu of constructs that have been associated with effective implementation and may be used in a range of applications [23, 24]. The five major domains of the CFIR are: (1) the intervention characteristics (includes evidence strength and quality, adaptability, and cost); (2) the outer setting (includes the economic, political, and social context within which an organization resides); (3) the inner setting (includes features of the structural, political, and cultural contexts through which the implementation process will proceed); (4) the characteristics of the individuals involved (includes knowledge and beliefs about the intervention, self-efficacy, and individual stage of change); and (5) the process by which implementation is accomplished (described next) [23, 24].

Eight constructs are related to the process of implementation and form the focus of this report. They consist of: (1) planning (developing schemes and tasks in advance); and (2) engaging (involving key individuals in the intervention). These key individuals include: (3) opinion leaders (individuals who have influence on their colleagues), (4) leaders (individuals with responsibility for implementing an intervention), (5) champions (individuals who drive through an implementation), and (6) change agents (individuals at an outside entity who influence decisions favorably). Finally, the last two process constructs are: (7) executing (accomplishing the implementation as planned); and (8) reflecting (feedback on progress and debriefing) [23, 24]. The aim of this study is to reflect on the process of recruiting racial/ethnic minority older adults for focus group discussions using the infrastructure of an existing community-based clinical outreach program and guided by the CFIR.

## Methods

### Context for the recruitment study

**Fig. 1:**
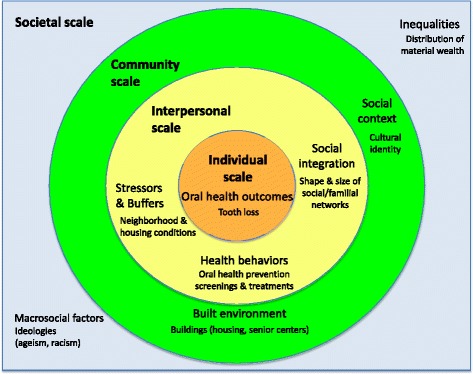
This simplified ecological model for thinking about pathways whereby social determinants at various scales (societal, community, and interpersonal) influence oral health and related health outcomes for both individuals and populations of older adults is adapted from a conceptual model titled, “Ecological model of social determinants of oral health for older adults” that first appeared in: Northridge ME, Ue F, Borrell LN, Bodnar S, De La Cruz L, Marshall S, Lamster IB. Tooth loss and dental caries in community-dwelling older adults in northern Manhattan. Gerodontology 2012; 29:e464-e473

The context for this recruitment research (in this case, our intervention) is our experience in conducting one of the social science components of an ongoing interdisciplinary study funded by the National Institute of Dental and Craniofacial Research (NIDCR) and the Office of Behavioral and Social Sciences Research (OBSSR) of the US National Institutes of Health (NIH). This larger study integrates social and systems science approaches to promote health equity, and is guided by the “Ecological model of social determinants of oral health for older adults.” A variety of methodological approaches (qualitative, quantitative, spatial, and systems science) have proved useful in thinking through pathways whereby social determinants at various scales influence oral health and related health outcomes, toward promoting healthy aging [25]. A simplified “Ecological model of social determinants of oral health for older adults” [25] is presented as Fig. 1, where selected factors at the societal, community, interpersonal, and individual scales are included that bear directly on oral health and related health outcomes for both individuals and populations of older adults*.*

The recruitment plan builds upon previous research and practice initiatives of the authors and their colleagues. Chief among these is the ElderSmile program, a community-based initiative sponsored by the Columbia University College of Dental Medicine to promote oral and general health and conduct health screenings in places where older adults gather, including senior centers [26, 27]. In an earlier NIH-funded study conducted with racial/ethnic minority older adults who attend senior centers in underserved urban neighborhoods, we found lower levels of tooth loss than in US national samples [25]. In order to learn from what is working well in this socioeconomically disadvantaged population, we obtained subsequent NIH funding to conduct social science research, including focus group discussions with racial/ethnic minority older adults intended to gather information about factors at the community, interpersonal, and individual scales toward enhancing community- and clinic-based service delivery and improving oral and general health outcomes for older adults.

### Eligibility criteria

To be selected to participate in the focus group discussions, participants (individuals involved) needed to meet the following criteria: (1) aged 50 years and older; (2) attended a senior center in northern Manhattan, New York, NY; (3) speak fluent English or Spanish; and (4) self-identify as African American, Dominican or Puerto Rican. To ensure geographic representation of northern Manhattan, approximately equal numbers of participants were recruited from senior centers in each of three northern Manhattan neighborhoods: Central Harlem (largely African American), Washington Heights/Inwood (largely Dominican), and East Harlem (largely Puerto Rican).

### Focus group segmentation

It is sound scientific practice to segment focus groups based on important characteristics that may influence either the issues discussed or the ability of the members to build rapport [28]. For analytic purposes, however, conclusions cannot be made on one or two groups per demographic segment. Thus, there was an inherent tension between simplifying the recruitment strategy and maximizing the amount of information that could be obtained with the fewest number of focus groups overall.

In the end, a total of 24 focus groups were conducted: 12 groups of men, and 12 groups of women. In each gender block, four groups were conducted with African Americans, four groups with Puerto Ricans, and four groups with Dominicans. Ten of the groups were conducted in English and 14 were conducted in Spanish (two of the Puerto Rican groups were conducted in English). Within each gender/racial/ethnic group, half of the groups were conducted with men or women who had visited a dentist in the past year, and the other half were conducted with men or women who had not visited a dentist in the past year (see Table 1).

**Table 1:** Numbers of focus groups conducted, segmented by race/ethnicity, language, gender, and time since last visited a dentist, New York, NY, 2013–2015

Characteristics used to segment focus groups (*N* = 24)		African American (*n* = 8)		Dominican (*n* = 8)		Puerto Rican (*n* = 8)	
		Spanish (*n* = 0)	English (*n* = 8)	Spanish (*n* = 8)	English (*n* = 0)	Spanish (*n* = 6)	English (*n* = 2)
Women (*n* = 12)	Last dental visit within the past year (*n* = 6)	0	2	2	0	2	0
	Last dental visit more than a year ago (*n* = 6)	0	2	2	0	1	1
Men (*n* = 12)	Last dental visit within the past year (*n* = 6)	0	2	2	0	1	1
	Last dental visit more than a year ago (*n* = 6)	0	2	2	0	2	0

The investigators elected to only include older adults who self-identified as African American, Dominican, and Puerto Rican, as these are the three largest racial/ethnic groups in northern Manhattan. It was decided that segmentation on the basis of cultural identity was important to build rapport among focus group members.

### Recruitment plan

Both field recruiters (implementation leaders) were bilingual (English/Spanish) and had several years of experience working with racial/ethnic minority older adults and senior center directors in northern Manhattan as the program staff for the ElderSmile network. The recruitment staff visited geographically diverse senior centers in northern Manhattan and directly approached older adults to explain the study, screen for eligibility, and solicit their participation in the focus groups (individuals involved). To achieve the desired group size of eight to ten participants, 14–16 potential participants were recruited to participate in each group. This over-recruiting was deemed necessary since focus groups frequently have poor attendance rates [29]. Based on field experience (executing the implementation), the recruitment plan was adapted by the study investigators and recruitment staff after engaged discussion of challenges posed and potential solutions at research team meetings (reflecting on the progress of the implementation), as explained next.

### Recruitment sites

The ElderSmile network of prevention centers were used as recruitment sites for focus group participants, and consisted of four center types: (1) Naturally Occurring Retirement Community; (2) US Department of Housing and Urban Development Section 202 Supportive Housing for the Elderly Program; (3) New York City Housing Authority; and (4) community senior or resource center [30]. No center director (opinion leader) contacted refused to allow focus group recruitment at any site, although the level of support offered during the recruitment period varied. According to the field recruiters, the most effective directors were “warm and welcoming” (engaging) and provided direct assistance to the recruitment staff, such as suggesting potential participants and opportune times for recruitment, making announcements asking for cooperation with the recruiters, and providing private space for screening whenever possible. We refer to these directors as change agents.

The populations of the centers also varied [30]. Some participants traveled by public transportation from the Bronx because they had been attending centers in Harlem but had moved; other centers drew participants from multiple Manhattan neighborhoods [30]. Certain centers were more racially/ethnically diverse than others, while some had younger cohorts of attendees [30]. On recruitment days, some centers were full, and some were virtually empty. Attendance was, of course, weather-related as well. Certain centers had attendees who were transitioning to care for the disabled or with cognitive difficulties who were not eligible for participation in the focus group discussions. Spaces within centers for making contact with and screening potential participants also varied, raising concerns around privacy and communication, especially with hearing impaired older adults.

### Focus group sites

Two venues (inner settings) were identified for the focus group sessions--one in Harlem and one in Washington Heights--to better ensure that participants did not need to travel more than a mile by provided taxis from their residential neighborhoods. After several months, however, all groups were held in the Washington Heights location, as the Harlem location proved logistically confusing for participants, since it was situated on the third floor of an office building. The focus group participants were delighted to learn that the Washington Heights location was a community space, which offered general health information via pamphlets and the use of computers to the public. Certain participants even inquired at the front desk about services offered or brought home reading materials.

## Results

Below we reflect on the process of recruiting racial/ethnic minority older adults for focus group discussions using the infrastructure of an existing community-based clinical outreach program, guided by the CFIR constructs that have been associated with effective implementation.

### Targets met

While the recruitment phase of the study was delayed by 6 months, the flexibility of the recruitment plan, the expertise of the research team members, the perseverance of the recruitment staff (implementation leaders), and the cultivation of change agents ultimately resulted in successfully executing the recruitment plan and meeting the study targets for enrollment in terms of both numbers of groups and numbers of participants (see Table 2). The original plan was to recruit participants, and then fill focus group slots. Nonetheless, when several months elapsed after determining the eligibility of older adults for the study, those recruited were sometimes no longer interested in participating or no longer available. Instead, the decision was made to recruit participants and fill focus group slots in an ongoing way. There was an initial 6-month delay in arriving at this solution, and then all of the focus groups were completed in the planned 2 years.

**Table 2:** Characteristics of participants in focus groups by race/ethnicity and for the total sample, New York, NY, 2013–2015

Characteristics		African American	Dominican	Puerto Rican	Total sample
	Participants	*n* = 72	*n* = 69	*n* = 53	*N* = 194
	Focus groups	*n* = 8	*n* = 8	*n* = 8	*N* = 24
Age (years)		mean = 68.3	mean = 71.6	mean = 68.5	mean = 69.5
	Standard deviation (SD)	SD = 10.2	SD = 9.6	SD = 10.0	SD = 10.0
		range = 50–92	range = 50–90	range = 50–91	range = 50–92
Age group	50–54	11.1% (8)	4.3% (3)	13.2% (7)	9.3% (18)
	55–59	6.9% (5)	1.4% (1)	7.5% (4)	5.2% (10)
	60–64	15.3% (11)	20.3% (14)	17.0% (9)	17.5% (34)
	65–69	20.8% (15)	15.9% (11)	11.3% (6)	16.5% (32)
	70–74	23.6% (17)	15.9% (11)	20.8% (11)	20.1% (39)
	75–79	8.3% (6)	21.7% (15)	18.9% (10)	16.0% (31)
	80–84	5.6% (4)	11.6% (8)	7.5% (4)	8.2% (16)
	85–89	4.2% (3)	5.8% (4)	0% (0)	3.6% (7)
	90 +	4.2% (3)	2.9% (2)	3.8% (2)	3.6% (7)
Gender					
	Male	44.4% (32)	49.3 (34)	45.3% (24)	46.4% (90)
	Female	55.6% (40)	50.7% (35)	54.7% (29)	53.6% (104)
Time of last dental visit					
	Within past year	54.2% (39)	59.4% (41)	47.2% (25)	54.1% (105)
	1–3 years ago	26.4% (19)	29.0% (20)	26.4% (14)	27.3% (53)
	More than 3 years ago	19.4% (14)	11.6% (8)	26.4% (14)	18.6% (36)
Primary language					
	English	100% (72)	0% (0)	18.9% (10)	42.3% (82)
	Spanish	0% (0)	98.6% (68)	49.1% (26)	48.5% (94)
	Both	0% (0)	1.4% (1)	32.1% (17)	9.3% (18)
Neighborhood of residence					
	Inwood	4.2% (3)	13.0% (9)	1.9% (1)	6.7 (13)
	Washington Heights	13.9% (10)	58.0% (40)	5.7% (3)	27.3 (53)
	East Harlem	15.3% (11)	5.8% (4)	79.2% (42)	29.4 (57)
	Central Harlem	30.6% (22)	4.3% (3)	5.7% (3)	14.4 (28)
	West Harlem	20.8% (15)	8.7% (6)	3.8% (2)	11.9 (23)
	Other	15.2% (11)	10.1% (7)	3.8% (2)	10.3 (20)

The ethnic groups did not differ significantly on any of the characteristics listed above, with the exception of primary language and neighborhood of residence, in accordance with the sampling strategy

**Fig. 2:**
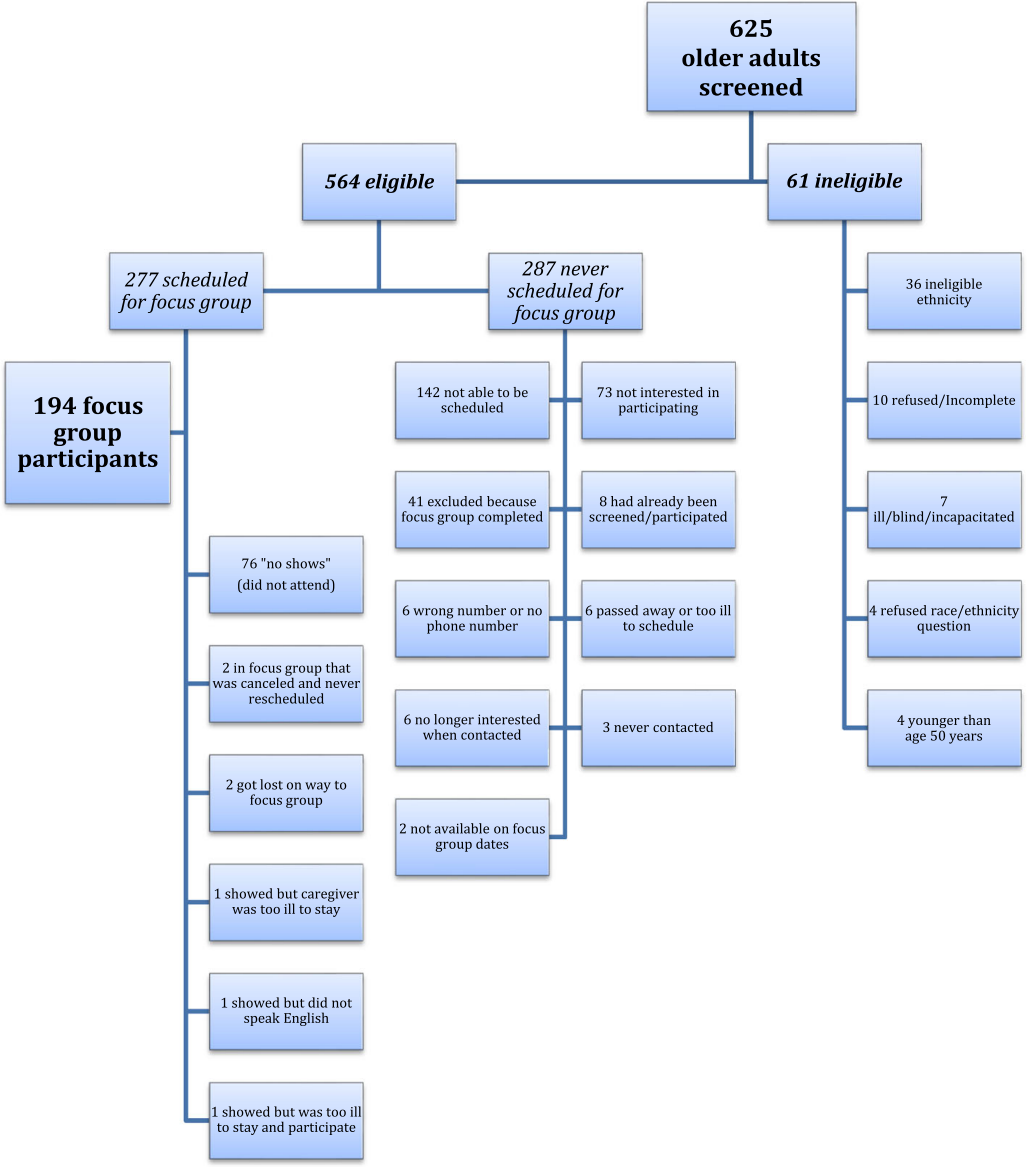
This flow chart details the screening and participation numbers of racial/ethnic minority older adults for a social science research study, as well as the reasons for ineligibility and nonparticipation in focus groups

The effort required to meet these targets was substantial. Of 625 older adults screened, a total of 194 African American, Dominican, and Puerto Rican older adults participated in 24 focus group discussions (see Fig. 2).

Potential demographic differences were examined between the 194 focus group participants and the 76 no show, the 142 not able to be scheduled, and the 79 not interested older adults (the 73 who were never interested were combined with the 6 who were not interested when contacted for a focus group to form a single not interested subgroup). Variables were created for each of these subgroups. Pearson’s chi-squared test was used to examine differences between the participants and the no show, not able to be scheduled, and not interested older adults, respectively. All calculations assume 2-sided tests with a significance level of alpha = 0.05. The only statistically significant difference between the participants and the no show older adults is that those without a dental visit in the past year were **more** likely to be no show older adults than were participants (*p* = 0.01). The only statistically significant difference between participants and the not able to be scheduled older adults is that adults who were 65 years or older were **more** likely to be not able to be scheduled older adults than were participants (*p* = 0.01).

On the other hand, there were several statistically significant differences between those who were not interested older adults and participants. Older adults who spoke both English and Spanish were **less** likely to be not interested than were participants (*p* = 0.05). African Americans were marginally (*p* = 0.06) **more** likely to be not interested than were participants. Those without a dental visit in the past year were marginally (*p* = 0.06) **more** likely to be not interested than were participants. Adults aged 65 years and older were **more** likely to be not interested than were participants (*p* = 0.03). Finally, Central Harlem residents were **more** likely to be not interested than were participants (*p* = 0.01). These differences are consistent with the experiences of the recruitment staff in filling focus group slots. Analyses are available upon request from the authors.

Analysis of the focus group discussions is providing important information for modeling activities toward improving community- and clinic-based service delivery and guiding policies to advance health equity for older adults. In addition, reflecting upon the implementation of the recruitment plan yielded the following insights for overcoming barriers to the enrollment of racial/ethnic minority older adults that may prove transferable or adaptable to other contexts.

#### Racial/ethnic identity

Originally, the screening instrument did not prompt participants to self-identify as African American, Dominican, or Puerto Rican per se, because we wanted participants to self-identify their races/ethnicities without biasing their designations, as per sound recruitment practice. Yet difficulties ensued. “Race/ethnicity is where we hit a bump,” in the words of one staff member (implementation leader), “especially with African Americans who felt they were Americans and didn’t need to define themselves as African Americans.” Frequently the response was turned back to the recruitment staff, with individuals asking, “What do *you* think I am?” The screener rejoinder advised recruitment staff to respond, “This is how *you* define yourself,” but this proved to be inadequate in certain situations. For instance, at one site a few women of Cuban descent became upset because the eligibility criteria were not stated at the outset of the screening process. They felt it was illegal to exclude them, and the center director (opinion leader) had to intervene to de-escalate the situation.

After this incident, we gained the advice of senior community-based colleagues (champions) and modified our screening procedure to make clear that we were only recruiting African American, Dominican, and Puerto Rican individuals, so that people could decline to be screened straight away. This successfully resolved the problem and no further incidents occurred. Participants self-identified as African American, Dominican, or Puerto Rican only; no participant self-identified as belonging to more than one of these groups.

#### Language

While all of the focus groups with Dominicans were conducted in Spanish, a substantial number of Puerto Ricans screened preferred speaking English. Rather than only conducting the Puerto Rican groups in Spanish as originally planned, we conducted two of the Puerto Rican groups in English (one with women and one with men). Still, many of the participants in the Dominican and Puerto Rican groups spoke both Spanish and English during the conversations held, so the facilitator needed to translate comments to ensure understanding among all group members.

#### Institutional mistrust

Another obstacle faced during the recruitment activities was the somewhat fraught historic relationships between the university and the surrounding communities (outer setting) due to the expansion of its academic and residential buildings in Central and West Harlem, and the consequent displacement of poor, mostly African American and Puerto Rican residents. While Puerto Ricans were more accepting of the university identification than the African Americans and Dominicans who resided nearby, they were also more concerned about participation in the study affecting their entitlements. In general, however, if any of the potential participants allowed the reading of the screener script, “They were interested.” Older adults who agreed to participate did so because the topic was of interest, they appreciated the $30.00 stipend, or they expressed interest in future benefits from the research to their communities at large. Those who refused to participate cited a lack of time, discomfort in group settings, or confusion about what a focus group was, even after it was explained as a *charla* (meaning discussion).

#### Gender

The original plan called for the recruitment staff to visit a prevention center and screen all attendees who were present. Certain groups were relatively easy to fill and participants were scheduled for a session soon after being screened, e.g., African American women without a dental visit in the past year. But other groups proved difficult to fill, e.g., all groups of men, and it would take considerably longer to find 15 or so individuals to over-recruit for a session with eight to ten participants. Unfortunately, after a few months, people would forget about the project, lose interest, change their contact information, or even pass away.

While outdoor recruitment was not part of the original recruitment plan, it was especially critical in recruiting African American men whose presence inside the centers was rare. The needed numbers were eventually reached by contacting men in parks and outside of neighborhood *bodegas* (meaning shops).

#### Targeted center recruitment

As the easier-to-reach participants were enrolled and the attendant groups were conducted, it became logistically advisable to shift from a geographically broad to a targeted center recruitment plan to enroll the subpopulations that remained. This was a less desirable sampling strategy scientifically because many of the individuals from, e.g., a Dominican group, were then recruited from a center that was heavily Dominican, and the group was then less likely to include Dominicans from centers that were predominantly comprised of other races/ethnicities or located in other neighborhoods. While their experiences might have differed from those in centers that included mostly people like themselves, we adapted the original recruitment plan in order to meet our targeted enrollment numbers and preferred group sizes.

#### Transportation

Once individuals confirmed attendance for a particular group, the recruitment staff coordinated pick-ups at centers and homes by a community taxi company. This transportation strategy was crucial in assuring focus group attendance, especially for older adults with mobility problems.

#### Meals

Although the afternoon sessions were usually held after the participants were provided lunch at the centers and may not have been especially hungry, hot meals were ordered from a local restaurant and were appreciated by the attendees, who often packed up the remaining food afterwards and brought it home. Sharing a meal together also served as an effective ice breaker for the sessions.

## Discussion

Here we reflect on the process of recruiting racial/ethnic minority older adults for focus group discussions using the infrastructure of an existing community-based clinical outreach program and explicitly link our findings to the constructs of the CFIR. In addition to the cultural barriers reviewed at the outset of the paper and addressed in this recruitment study through the involvement of experienced, bilingual (English/Spanish) field recruiters, attempts to recruit older adults may be hampered by the physiologic changes of aging and the health issues they face. These include hearing and visual impairment, fatigue, limited mobility, cognitive slowing, and chronic conditions [13, 31, 32]. Age differences between participants and researchers may also serve as barriers, since older adults may be reluctant to share information with young people [10].

Practical solutions to potential health-related barriers among older adults include tailoring the pacing of the recruitment presentation, [14] using large-print materials, [33] and providing appropriate breaks and nourishment [18]. The health concerns of older adults may be incorporated into the recruitment plan by providing education around the condition being studied and the potential benefits to the larger society and science of participating in the research [18]. Providing services such as health screenings may be apt in demonstration projects of new models of care [9, 10, 34].

Other recommendations include having a proactive, detailed recruitment plan [13]. Research has also found consumer-centered or social-marketing recruitment strategies to be particularly effective in this population [19, 34, 35]. Proactive face-to-face recruitment has been found to be more effective than relying on passive methods such as informing the community through public notices and waiting for volunteers to call [5, 12, 14, 33]. As older adults may have difficulties with mobility or limited time due to other responsibilities, addressing transportation needs, [31] or recruiting where older adults gather or reside, e.g., senior centers, [11] Medicare PACE programs, [36] or their residences [9] rather than at academic institutions may be more effective [18]. Perhaps unsurprisingly, principal investigators (leaders) who report valuing racial/ethnic minority inclusion found more success in recruiting them than those who do not report valuing their inclusion [37]. Studies among both older [31] and minority [6] research participants highlight the importance of altruism; thus, highlighting the benefit of their participation to their larger community may motivate participation [14].

It is important to recognize heterogeneity among racial/ethnic minority older adults, [20] such that there is no “one size fits all” approach [21]. In addition to issues of race/ethnicity and age, concerns about research participation have also been found to vary by gender [38]. There is a need for empirical studies on intersectional components of identity and research relationships [39].

The limitations of this study include that older adults were recruited from senior centers and other places where older adults gather in northern Manhattan. Hence, the findings may not be generalizable to older adults who are institutionalized or living in other locales. Further, the recruitment plan leveraged trained recruitment staff and relationships with senior center directors that were fostered through a community-based program to promote oral and general health for older adults. Other research teams may lack previous engagement with key individuals who may usefully guide implementation of their recruitment plans (e.g., opinion leaders in community sites, program staff as implementation leaders, experienced community-based researchers as champions, and influential directors of community-based organizations as change agents).

## Conclusions

The extensive literature on participant recruitment for research is largely focused upon clinical research and institution-based recruitment. This study has provided further insights into the factors affecting community-based recruitment of a challenging and challenged population for social science research: racial/ethnic minority older adults. Our experience detailed here adds to the available literature on the topic of recruitment of racial and ethnic minority older adults for focus group discussions in two important ways. First, we reported on the experiences of recruitment through community sites or “third places” which leveraged the social and physical infrastructure of an existing program. Second, we used the CFIR to guide the critical appraisal of the recruitment process, which underscored important considerations for both reaching and engaging this underserved population, especially in terms of understanding the disparate roles of the individuals involved in implementing and facilitating the recruitment plan.

Many issues identified in the literature were also important to our experience: a historical perspective; the heterogeneity of the populations involved; gender differences; cultural sensitivity; trust; understanding recruitment messages; physical health and cognitive difficulties; and altruism. Given the increasing older adult population in the United States and around the world, this recruitment study appraisal may usefully inform future considerations for effective inclusion of racial/ethnic minority older adults in health and social science research.

## Abbreviations

CFIR: Consolidated Framework for Implementation Research
NIDCR: National Institute of Dental and Craniofacial Research
NIH: National Institutes of Health
OBSSR: Office of Behavioral and Social Sciences Research
